# The Surgery, Surgical Pathology and Surgical Anatomy of the Female Pelvic Organs

**Published:** 1880-07

**Authors:** 


### BIBLIOGRAPHICAL.

THE SURGERY, SURGICAL PATHOLOGY AND SURGICAL ANATOMY OF THE FEMALE RELVIC ORGANS. In a series of plates taken from nature, with commentaries, notes, and cases, by Henry Savage, M.D., London, Fellow of the Royal College of Surgeons of England, one of the consulting Medical Officers of the Samaritan Hospital for women. Third edition revised and greatly extended—32 plates and 22 wood engravings, with special illustrations of operations on Vesico-Vaginal fistula, ovaritomy and perineal operations. New York: Wm. Wood & Co., 27 Great Jones street, 1880.

This is one of the volumes of "Wood's Library of Standard Medical Authors," and consists, to a large extent, of the anatomy and pathological anatomy of the female pelvic organs. It will be found useful, therefore, to those who wish familiarity with these parts, in order to the diagnosis and proper surgical treatment of their diseases.

